# Tracheal stenting by rigid bronchoscopy in right lateral decubitus position in an awake patient^☆^

**DOI:** 10.1016/j.heliyon.2023.e18678

**Published:** 2023-07-27

**Authors:** Clarissa B. Smith, Lucas Pitts, Maykol Postigo

**Affiliations:** a The University of Kansas Health System, Department of Internal Medicine, Mailstop 1022 3901 Rainbow Boulevard, Kansas City, KS, 66160, United States; b The University of Kansas Health System, Division of Pulmonary, Critical Care, and Sleep Medicine, United States

**Keywords:** Interventional pulmonology, Rigid bronchoscopy, Central airway occlusion, Tracheal stent, Dexmedetomidine

## Abstract

Rigid bronchoscopy is a common procedure for central airway obstructions (CAO). Many patients with advanced lesions causing CAO have tenuous, positionally dependent respiratory status which requires additional procedural considerations. This case report describes a 57-year-old man with high grade epithelioid angiosarcoma of the right lung and pleura who underwent placement of a tracheal stent by rigid bronchoscopy in the novel procedural conditions of right lateral decubitus, semi-sitting position with dexmedetomidine, midazolam, and propofol for moderate sedation. Dexmedetomidine, which is currently in use for flexible bronchoscopy due to its analgesic, anxiolytic, and antisialogogue properties performed ideally and should be further evaluated for this indication.

## 1 Introduction

Rigid bronchoscopy is a common procedure for central airway obstructions (CAO). Typically rigid bronchoscopies are performed under general anesthesia or utilizing deeply sedating intravenous medications including hypnotics such as benzodiazepines, analgesics commonly opioids such as fentanyl and remifentanil, and neuromuscular blocking agents. Because the rigid bronchoscope is uncuffed, specialized ventilation strategies must be used, most commonly high-frequency jet ventilation [1,2]. Many patients with advanced lesions causing CAO have tenuous, positionally dependent respiratory status which requires additional procedural considerations such as alternative positioning and modes of ventilation including apneic oxygenation, spontaneous assisted ventilation, closed system ventilation, or manual jet ventilation [2].

## 2 Case vignette

**Fig. 1:**
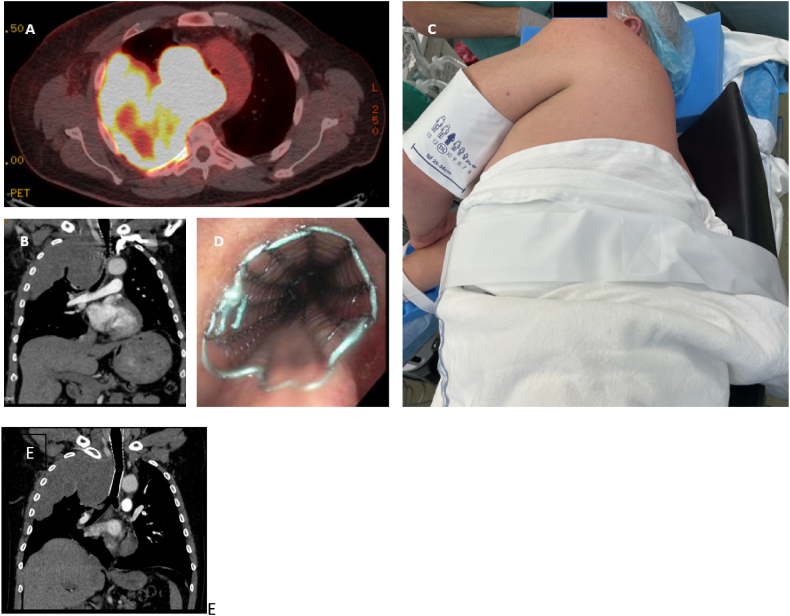
A. Positron Emission Tomography (PET) of heterogeneously enhancing right-sided pleural-based masses invading adjacent mediastinum and compressing adjacent mid trachea, esophagus, and superior vena cava; B. Computed tomography (CT) sagittal view demonstrating severe mid tracheal compression; C. Patient in right lateral semi-seated position for rigid bronchoscopy; D. 16 × 60°mm Ultraflex metallic stent deployed in the distal trachea; E. Post procedural CTA with patent tracheal stent.

A 57-year-old man with recently diagnosed high grade epithelioid angiosarcoma involving the right lung and pleura presented through the ED for acute on chronic hypoxemic respiratory failure and chest pain. He was in severe respiratory distress only tolerating tripod or semi-sitting position leaning on his right side with 3L of supplemental oxygen. Review of systems was significant for intermittent hemoptysis and right sided facial and neck edema. Computed Tomography (CT) with contrast showed extensive right heterogeneously enhancing pleural-based masses with evidence of rapid progression since outside imaging including invasion of adjacent mediastinum, compression of adjacent mid trachea, esophagus, and superior vena cava (SVC) (Fig. 1A and B).

After a multidisciplinary discussion the decision was made to address the SVC, after which he was taken to the bronchoscopy suite for placement of a tracheal stent. Due to the patient’s intolerance of supine positioning and high risk for general anesthesia due to central airway occlusion (CAO), he was placed in the right lateral semi sitting position (45°) with moderate sedation consisting of midazolam 2°mg and dexmedetomidine (DEX) between rate of 1.2–1.8°mcg/kg/h for a total of 40°mcg (Fig. 1C). Nebulized tetracaine 0.25%/Epinephrine 0.003% was given pre-procedurally. A flexible bronchoscope was introduced through the mouth and into the trachea where 10mL tetracaine 0.25%/Epinephrine 0.003% was injected transtracheally and also liberally applied topically. Significant tracheal deviation/narrowing due to external compression of the trachea was observed. There was a 5°cm long segment of tracheal narrowing secondary to external compression with a lumen around 4°mm in the distal end of the trachea. Ahead of placement of rigid bronchoscope, 20°mg propfol was given intravenously. While still breathing spontaneously, a 16 × 60°mm Ultraflex metallic stent was deployed in the distal trachea with significant improvement of tracheal diameter to 70% of normal (Fig. 1D). The patient tolerated the procedure without complication and noted immediate improvement in his respiratory status including tolerating a supine position (Fig. 1E).

The patient recovered from bronchoscopy uneventfully with significantly improved respiratory status. He received inpatient external beam radiotherapy to the mediastinal mass in five sessions of 6°gy (30°gy total) and was discharged to home with scheduled oncologic follow-up for initiation of cytotoxic chemotherapy. At pulmonology follow-up one-month post-procedurally, the stent remained in place radiographically and the patient was symptomatically stable on 4 L supplemental oxygen. He has tolerated two cycles of gemcitabine and docetaxel with plans for additional cycles as tolerated and additional pulmonology follow-up six weeks after initiation.

## 3 Clinical discussion

This patient’s case required a novel set of procedural conditions to balance operative risks and improve toleration of rigid bronchoscopy (RB) given his severe respiratory distress and positional restriction from CAO.

Right lateral semi-seated position was employed due to airflow mechanics. While it is not unusual to pursue lateral decubitus positioning in flexible bronchoscopy (FB) to safely improve toleration, reduce atelectasis, and improve accessibility of certain targets, use in RB has not been previously described in the literature [3,4]. Lateral rotation of the head is used to manipulate the positioning of the RB after rigid intubation.

While RB has become increasingly innovative, many conventions including general anesthesia have remained standard [5]. We chose RB over FB to deploy this patient’s stent based on degree of tracheal compression, distal location, need for direct visualization for placement of the stent, and need for a large working channel in case of decompensation. Given the high risk of airway collapse during induction of general anesthesia in CAO and anticipation of alternation between flexible and rigid bronchoscopes, we favored moderate sedation with spontaneous ventilation for this patient’s procedure. The patient was counseled extensively regarding risks and alternatives as well as consented for possible backup plans.

## 4 Review of the literature

Dexmedetomidine (DEX) is an alpha-2 adrenoreceptor agonist with analgesic, anxiolytic, and antisialogogue properties known to preserve the respiratory drive [[6], [7], [8]]. It has been increasingly recognized for moderate procedural sedation in FB with several benefits compared to midazolam and propofol including fewer episodes of hypoxia [9,10].

Bronchoscopists have widely recognized the potential benefits of DEX in FB. One study reported that patients undergoing FB who received DEX, propofol, and fentanyl had a statistically significant reduction in procedural interruptions due to cough or movement as well as improved bronchoscopist satisfaction compared to those who received midazolam, propofol, and fentanyl [11]. A prospective evaluation of FB under moderate sedation using DEX and fentanyl versus propofol and fentanyl demonstrated comparable number of times topical anesthetic was required for coughing as well as comparable qualitative patient and bronchoscopist evaluation [10].

Evaluation of DEX’s performance has been extended to awake fiberoptic intubations. Bergese et al. described four challenging airways in which awake fiberoptic intubations were completed using DEX including one in which DEX was the only agent employed [8].

Though awake RB for foreign body removal is standard practice in the pediatric population, it has only recently been reported in adults. The initial indication attempted was tracheal or bronchial fistula with the aim of maintaining spontaneous ventilation. Mieda et al. described two such cases which were induced with general anesthetic sevoflurane and in one case DEX was used adjunctively without episodes of hypoxia or complication [12]. In the other case, adjunctive remifentanil was given, however propofol was additionally required to suppress cough reflex [12]. Other reports describe RB under intravenous-only procedural sedation including a series of 79 procedures with two performed using DEX alone [13]. Another case series describes three patients with malignant tracheal or bronchial fistulae who successfully underwent awake rigid bronchoscopy for stenting with spontaneous ventilation utilizing DEX and remifentanil alone except in one case which required propofol boli to suppress cough reflex [14]. RB for central airway occlusion under spontaneous ventilation using DEX in combination with ketamine was also reported as ‘near ideal’ in terms of oxygenation, hemodynamics, and procedural sedation [15].

In our patient’s case, advanced disease and severe respiratory compromise required both pharmacologic and anatomic procedural considerations. With the use of DEX and a lateral semi-seated position, the patient safely tolerated tracheal stenting under spontaneous respiration without hypoxia. DEX has a role in the bronchoscopy suite, not only in FB but also for its demonstrated efficacy in RB as well and additional investigations are needed to further describe its indications.

## Declaration of competing interest

The authors declare that they have no known competing financial interests or personal relationships that could have appeared to influence the work reported in this paper
